# Subject-Specific Head Model Generation by Mesh Morphing: A Personalization Framework and Its Applications

**DOI:** 10.3389/fbioe.2021.706566

**Published:** 2021-10-18

**Authors:** Xiaogai Li

**Affiliations:** Division of Neuronic Engineering, Department of Biomedical Engineering and Health Systems, KTH Royal Institute of Technology, Stockholm, Sweden

**Keywords:** finite element modeling, personalized simulation, traumatic brain injury, brain stimulation, neuroimage registration, biomechanics

## Abstract

Finite element (FE) head models have become powerful tools in many fields within neuroscience, especially for studying the biomechanics of traumatic brain injury (TBI). Subject-specific head models accounting for geometric variations among subjects are needed for more reliable predictions. However, the generation of such models suitable for studying TBIs remains a significant challenge and has been a bottleneck hindering personalized simulations. This study presents a personalization framework for generating subject-specific models across the lifespan and for pathological brains with significant anatomical changes by morphing a baseline model. The framework consists of hierarchical multiple feature and multimodality imaging registrations, mesh morphing, and mesh grouping, which is shown to be efficient with a heterogeneous dataset including a newborn, 1-year-old (1Y), 2Y, adult, 92Y, and a hydrocephalus brain. The generated models of the six subjects show competitive personalization accuracy, demonstrating the capacity of the framework for generating subject-specific models with significant anatomical differences. The family of the generated head models allows studying age-dependent and groupwise brain injury mechanisms. The framework for efficient generation of subject-specific FE head models helps to facilitate personalized simulations in many fields of neuroscience.

## Introduction

Finite element (FE) head models have become powerful tools to simulate brain stimulations with direct current (tDCS) (Datta et al., 2009; Datta et al., 2012; Huang et al., 2013; Windhoff et al., 2013; Opitz et al., 2015; Alekseichuk et al., 2019; Li et al., 2020; Wang et al., 2020), magnetic (TMS) (Opitz et al., 2013), and ultrasound (TUS) (Legon et al., 2014). Such models are also being used to study the development of neurodegenerative diseases (Fornari et al., 2019; Noël and Kuhl, 2019; Weickenmeier et al., 2019) and biomechanical consequences of neurosurgery (Weickenmeier et al., 2017; von Holst and Li, 2014; Li et al., 2015; Ji et al., 2009; Hu et al., 2007; Miller et al., 2010). In particular, FE head models have been tremendously used to study traumatic brain injuries (TBIs) in the last decades (see reviews (Giudice et al., 2019; Horstemeyer et al., 2019; Madhukar and Ostoja-Starzewski, 2019)). Meshing is a first step in generating FE models by discretizing a continuous domain into a finite number of elements, e.g., tetrahedral or hexahedral elements. Generation of FE head models is often time-consuming and challenging due to its complex geometry, though tetrahedral elements are relatively easier to generate, e.g., automatic pipelines have been reported to efficiently generate tetrahedral head models for simulating brain stimulations (Huang et al., 2013; Windhoff et al., 2013). Such efficiency is partially attributed to the well-developed automatic tetrahedral meshing algorithms within mathematics and computer science (Baker, 2005); it is also because the involved partial differential equations (PDEs) are less computationally demanding, permitting a huge number of tetrahedral elements (up to >10 million (Datta et al., 2009)) to capture anatomical details of the brain. Thus, personalized simulations with anatomically detailed subject-specific head models are largely facilitated in these brain stimulation fields.

In contrast to tetrahedrons, hexahedral elements are much more challenging to generate (Baker, 2005; Shepherd and Johnson, 2008) but are preferred in FE head models intended for studying TBIs (hereafter called head injury models) due to their higher efficiency for simulating the incompressible brain under impact (Li et al., 2021). Furthermore, the involved PDEs in head impacts consist of geometrical, material nonlinearity, and complex contacting algorithms, which are more computationally demanding. As a result, many state-of-the-art head injury models (Zhang et al., 2001; Horgan and Gilchrist, 2003; Kleiven, 2007; Takhounts et al., 2008; Mao et al., 2013a; Sahoo et al., 2014; Ji et al., 2015a; Atsumi et al., 2016) use hexahedrons despite the meshing challenges and have simplified brains to reduce the number of elements for computational efficiency. For example, these models have smoothed out brain surfaces and do not have sulci, and gyri, resulting in fewer elements (often <1 million). While a simplified representation of the brain is a reasonable trade-off for computational efficiency, it’s also partially due to the challenges for meshing techniques (e.g., the blocking technique (Mao et al., 2013b)) to capture the anatomical details. It is worth mentioning that the voxel approach is efficient in generating hexahedrons by converting image voxels to hexahedral elements, either directly or with smoothing algorithms. However, a known concern is a less accurate peak strain/stress predicted from such models, especially on the surfaces due to jaggedness. Nevertheless, careful choice of sufficiently refined mesh and result analysis allow such models to provide valuable insights due to their anatomical accuracy (see discussion in (Li et al., 2021)). Besides the much less developed automatic algorithms for generating hexahedrons (Baker, 2005; Shepherd and Johnson, 2008), a necessity to include falx and tentorium to account for their important structural influence on brain mechanical responses during impact (Ho et al., 2017) poses an additional challenge for subject-specific head injury model generation while both structures are often neglected in head models for simulating tDCS, TMS, and TUS. A detailed analysis of the current meshing challenge for head injury models is found in a previous study (Li et al., 2021).

Therefore, the generation of FE head injury models with anatomical details remains a challenge and has become a bottleneck hindering personalized simulations. FE head models without anatomical details such as sulci and gyri also hinder studying detailed mechanisms at areas of interest, such as chronic traumatic encephalopathy (CTE) with pathologies observed at sulcal depth (McKee et al., 2015). Studies have also shown that the brain size/shape influences brain mechanical responses significantly under impact (Kleiven and von Holst, 2002; Li et al., 2021), suggesting the importance of using personalized models to study the onset of TBI in real life. Along with the many existing adult healthy FE head models, there are only a few elderly, children, and infant models (e.g., (Li et al., 2011; Giordano et al., 2017; Li et al., 2017; Li and Kleiven, 2018; Hajiaghamemar et al., 2019; Li et al., 2019; Zhou et al., 2019; Zhou et al., 2020)). TBIs are influencing all age groups, especially infants and the elderly are overrepresented (Pedersen et al., 2015). Thus, it is imperative to investigate efficient approaches for generating detailed subject-specific head injury models across the lifespan and for pathological brains to understand the injury mechanisms and develop preventions.

This study addresses the challenge of generating subject-specific head injury models with hexahedrons, especially concerns about mesh morphing, which is an efficient approach for generating subject-specific models. The approach has been used in many biomechanics fields on different organs (Couteau et al., 2000; Castellano-Smith et al., 2001; Fernandez et al., 2004; Sigal et al., 2008; Bucki et al., 2010; Bijar et al., 2016; Park et al., 2017), full-body models (Davis et al., 2016; Beillas and Berthet, 2017; Liu et al., 2020), as well as for detailed (Giudice et al., 2020; Giudice et al., 2021; Li et al., 2021; Montanino et al., 2021) and simplified brain models (Hu et al., 2007; Ji et al., 2011; Ji et al., 2015b; Wu et al., 2019). A typical procedure involves image registration (rigid or affine and followed by nonlinear registrations), from which a displacement field representing the geometrical difference between the subject and baseline model is obtained. The displacement field is then applied to morph the baseline model, resulting in a personalized model with updated nodal coordinates while preserving element connections. The displacement field derived from image registrations should generally comply with continuum mechanics conditions on motion, requiring diffeomorphic, non-folding, and one-to-one correspondence to avoid excessive element distortions (Bucki et al., 2010).

In particular, deformable image registration-based mesh morphing has been applied to personalize detailed brain models of healthy subjects (Giudice et al., 2020; Giudice et al., 2021; Li et al., 2021; Montanino et al., 2021). However, despite intensive efforts, inter-subject registration between brains with significant anatomical differences is still challenging within neuroimaging field with limited registration accuracy (Kim et al., 2015). Moreover, when applying image registration for mesh morphing, there is a higher requirement on the smoothness of the obtained displacement field to ensure acceptable element quality in the morphed mesh. Therefore, one major challenge for using mesh morphing to generate subject-specific FE head models is how to design an image registration pipeline that leads to high registration accuracy, meanwhile, not causes excessive element distortions. In a previous study (Li et al., 2021), we proposed a hierarchical image registration pipeline that allows efficient generation of subject-specific head models for healthy adult subjects. But a pipeline that allows morphing a baseline model to subjects with significant anatomical differences is yet to be developed.

Thus, this study aims at developing a personalization framework capable of generating subject-specific head models across the lifespan and for pathological brains with significant anatomical changes. The framework consists of hierarchical multiple feature and multimodality image registration pipelines, mesh morphing, and mesh grouping. Six subject-specific head models are generated to demonstrate its capacity, including a newborn, 1Y, 2Y, adult, 92Y, and a hydrocephalus brain. The results show that the framework is robust to generate subject-specific models across the lifespan and for pathological brains with significant anatomical changes by morphing a baseline model. This framework helps to facilitate personalized simulations in many fields within neurosciences, especially for studying TBIs in which personalized simulations are hindered due to the meshing challenge.

## Materials and Methods

### Subjects

**FIGURE 1:**
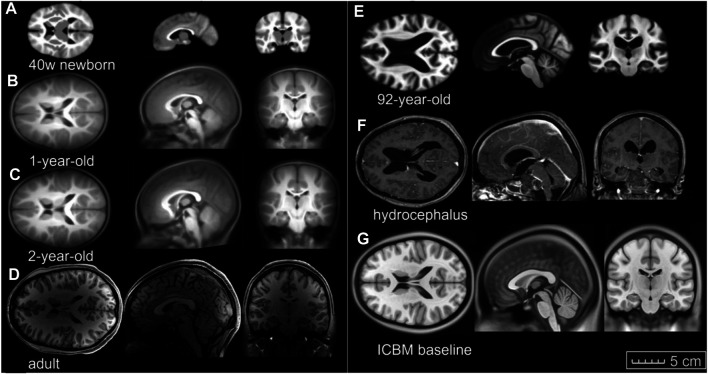
Image data used in this study. Axial, coronal, and sagittal views of **(A)** 40-week-old newborn, **(B)** 1-year-old, **(C)** 2-year-old, **(D)** an adult, **(E)** an elderly of 92-year-old, **(F)** hydrocephalus brain, and (**G**) the ICBM baseline (the same length scale applies).

Images of the six subjects (Figures 1A–F) are acquired from previously published open-access datasets, except the hydrocephalus brain is from the author’s previous study. The baseline ICBM image (Figure 1G) corresponds to the baseline head model. For detailed preprocessing steps for these images, the readers are referred to the original studies. A brief description is found below and summarized in Table 1.

• Images of a newborn (denoted as 0Y afterward), 1Y, and 2Y are obtained from the UNC Infant 0-1-2 atlases (Shi et al., 2011) constructed based on 95 subjects with complete 0-1-2Y longitudinal scans of T1W and T2W images acquired with a 3T MRI scanner. Each atlas consists of T1W images, tissue probability maps, and anatomical parcellation maps.
• Image of a single subject from the WU-Minn HCP database in the 26–30 age group, including T1W and T2W images, was acquired with a 3T MRI scanner (Van Essen et al., 2013).
• Image of an elderly (92Y) from the Brain Imaging of Normal Subjects (BRAINS) atlas was created from 48 healthy elderly subjects within age group 9193Y as detailed by Dickie et al. (2016). The atlas contains T1W and tissue probability maps.
• Image of a hydrocephalus subject with a mass lesion at the brain stem front is reused from a previous study (Li and von Holst, 2013).
• The 1-mm isotropic ICBM 2009c Nonlinear Symmetric template (Fonov et al., 2009; Fonov et al., 2011) was constructed based on T1W images from 152 subjects between 18.5–43.5Y acquired on a 1.5 T MRI scanner.

**TABLE 1:** Subjects involved in this study.

Subject	ICV (ml)	Imaging modality used for registration	Image sources
0-year-old^a^	463	T1W (atlas)	Shi et al. (2011)
1-year-old	1,015		
2-year-old	1,274		
adult	1,480	T1W, T2W (single subject in age group “26–30”)	Van Essen et al. (2013)
92-year-old^b^	1,323	T1W (atlas)	Dickie et al. (2016)
Hydrocephalus	1,255	T1W (single subject)	Li and von Holst, (2013)
ICBM baseline	1,885	T1W, T2W (atlas)	(Fonov et al., 2009; Fonov et al., 2011)

a The cerebellum in the T1W atlas was stripped in the original database. The available T2W atlas has a cerebellum but is not chosen in this study as per the requirement of the pipeline.

b Atlas of age group 91-93Y denoted as 92Ys throughout this study for simplicity.

### Baseline FE Head Model

**FIGURE 2:**
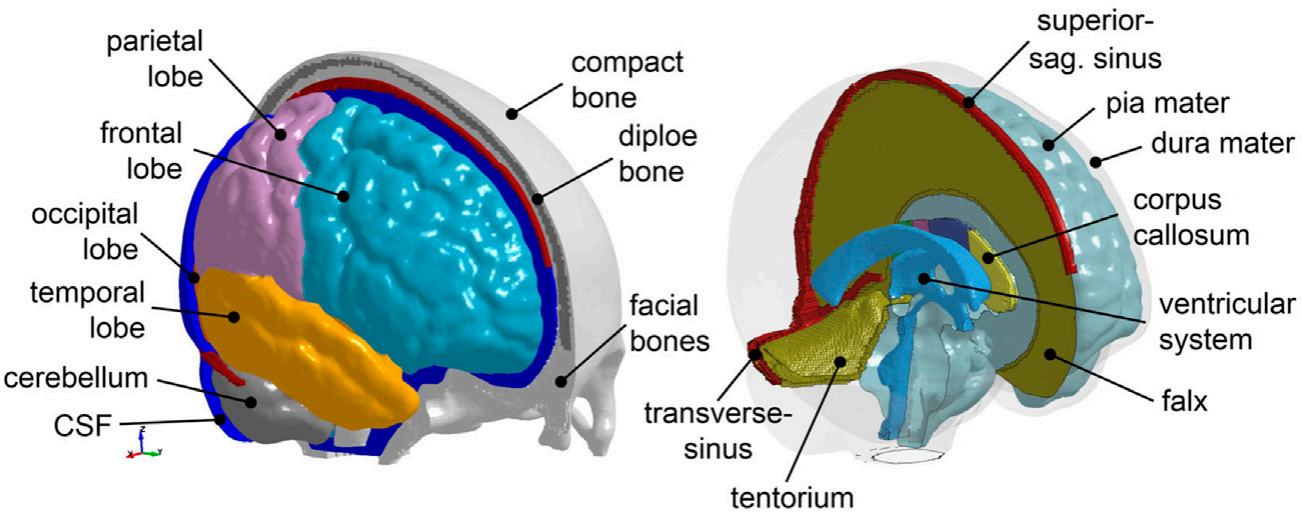
The baseline ADAPT head model with major components illustrated. The meshes are not shown for a better illustration.

A previously developed FE head injury model (the ADAPT model) (Li et al., 2021) serves as a baseline in this study, which is morphed to obtain subject-specific head models. The ADAPT model has been generated based on and has the same geometry as the ICBM template. The model includes the brain, skull, meninges, CSF, and superior sagittal sinus (SSS) (Figure 2). The brain is divided into primary structures of cerebral gray matter (GM) (i.e., cerebral cortex), cerebral white matter (WM), corpus callosum (CC), brain stem (BS), cerebellum GM and WM, thalamus, and hippocampus. The cerebrum is further divided into frontal, frontal, parietal, temporal, and occipital lobes; CSF is divided into outer CSF and ventricular system including lateral ventricles and 3^rd^ and 4^th^ ventricles connected by the cerebral aqueduct. Continuous mesh is used between brain components throughout the model. The total number of elements in the head model is 4.4 million hexahedral and 0.54 million quad elements. The minimum Jacobian in the brain is 0.45. The brain is modeled as hyper-viscoelastic material to account for large deformations and strain rate dependence of the tissue. Pia, dura/falx/tentorium are modeled with nonlinear hyperelastic material using simplified rubber/foam based on the average stressstrain experimental data (van Noort et al., 1981; Aimedieu and Grebe, 2004). The model has been validated against experimental data of close to or injury level brainskull relative motion, brain strain, and intracranial pressure. Details of the model development, validation, and capacity to study brain responses under impact are presented earlier (Li et al., 2021).

### Personalization Framework for Subject-specific Head Model Generation

**FIGURE 3:**
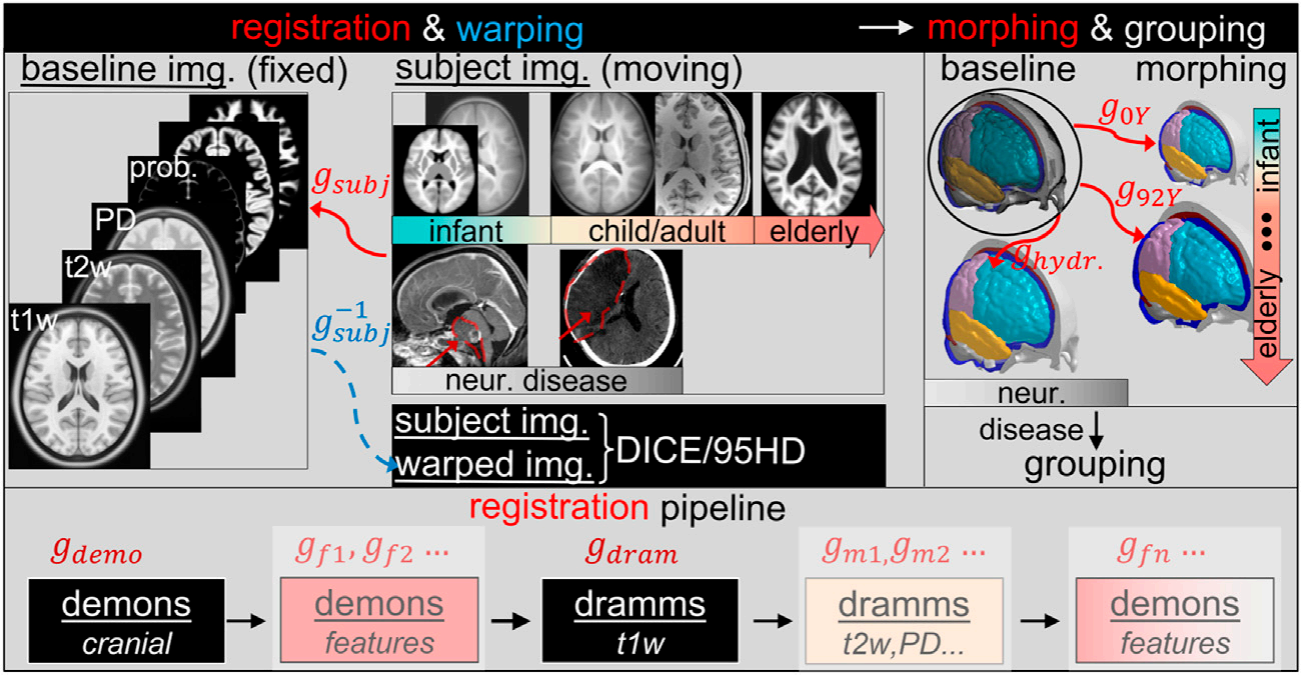
Overview of the personalization framework.

The personalization framework consists of image registration pipelines, mesh morphing, and mesh grouping (Figure 3). Image registration is an essential part of the framework. A complete registration pipeline involves hierarchical registrations with multiple features and multimodality images shown at the lower row of Figure 3. The sum of dense displacement fields obtained from each registration step is used to morph the baseline head model to obtain subject-specific models. Afterward, the WM of the morphed brain is regrouped according to the segmented WM image mask of the subject, resulting in the final subject-specific model. Details of each component of the framework are presented in the following subsections.

#### Registration Pipeline With Multiple Features and Multimodality Imaging

A complete registration pipeline contains five steps (Figure 3 lower row). First, Demons registration is performed between the segmented cranial masks of the baseline ICBM (corresponding to the baseline ADAPT head model) and the subject after being rigidly aligned, resulting in a transformation, i.e., dense displacement field $g_{demo}$ . Second, Demons registration of *features* is performed, obtaining $g_{f1},g_{f2}\ldots$ . Third, Dramms registration is performed on T1W images inherited from Demons steps, obtaining $g_{dram}$ . Next, Dramms registration is performed with multimodality images, obtaining $g_{m1,}g_{m2}\ldots$ . Finally, brain lesions are handled by more Demons feature registration steps, obtaining $g_{fn}\ldots$ . In all registration steps, the subject’s image serves as *moving* image, and the baseline ICBM image serves as *fixed* image. Note that *features* in this study refer to the segmented binary images of anatomical regions such as lateral ventricles, corpus callosum, or lesion. The input images to Demons registration steps are segmented binary masks. Thus, these steps capture local anatomical changes between the *moving* and *fixed* images only in size and shape, while subsequent Dramms registrations capture the internal anatomical differences within the volumes of the binary image masks. The practical usage of the registration pipeline is demonstrated in *Application of the Framework for Subject-specific Model Generation* with six subjects.

In particular, for all Demons steps described above, the diffeomorphic Demons registration algorithm (Vercauteren et al., 2009) implemented in the open-source software *Slicer 3D* is used. Dramms registration algorithm (Ou et al., 2011) implemented as open-source code by the authors (Dramms version 1.5.1, 2018) is used on MRI images of different modalities. Note that for all the six subjects, a smoothness weight, i.e., the *-g* option (see DRAMMS Software Manual), is always set to 1.0 in Dramms registration to ensure a smooth displacement field.

#### Morphing

The sum of dense displacement fields from all registration steps (Eq. 1) represents the anatomical differences between the subject and the baseline ICBM images.

$$g_{subj}=g_{demo}+g_{f1}+g_{f2}\ldots +g_{dram}\ldots +g_{m1}+g_{m2}\ldots \;+g_{fn}.$$ (1)

As the baseline, ADAPT model is in the same space as the ICBM image; thus, applying $g_{subj}$ to the baseline head model (Figure 2) leads to a subject-specific head model of the subject. For this, the following step is performed to morph the nodes of the baseline head model to new positions:

$$x^{i}=X^{i}+u^{i},$$ (2)

where $X^{i}$ is the nodal coordinate of node i , $u^{i}\;$ is the linearly interpolated displacement vector at node n from $g_{subj}$ , $x^{i}$ is the updated nodal coordinate, together with the same element definitions as the baseline, forming a subject-specific head model.

#### Grouping of WM

To capture the subject’s WM, the morphed brain elements are regrouped based on the segmented binary image of the subject’s cerebral WM. This is achieved by assigning brain FE elements as WM based on Cartesian coordinates of the segmented WM voxels with the following procedures:

- For each element, all WM voxels inside or intersect to a single element of the brain are identified based on spatial coordinates.
- The eight vertices and one centroid of each voxel (i,j,k) are judged; vertices gain a weight of one if falling inside the element; the centroid gains a weight of two if falling inside the element. Weights of the eight vertices and the centroid of the voxel add up, resulting in a total weighting factor for each voxel $w_{i,j,k}$

$$w_{i,j,k}=\overset{9}{\underset{m=1}{\sum }}w_{i,j,k}^{m}$$. (3)

- Finally, weights of each voxel belong to the same label (e.g., the segmented binary image with label A) added up, obtaining a final weight factor for each label. The element is grouped to the label with the largest weight.

$$w_{A}=\underset{\left(i,j,k\right)\in A}{\sum }w_{i,j,k}.$$ (4)

**FIGURE 4:**
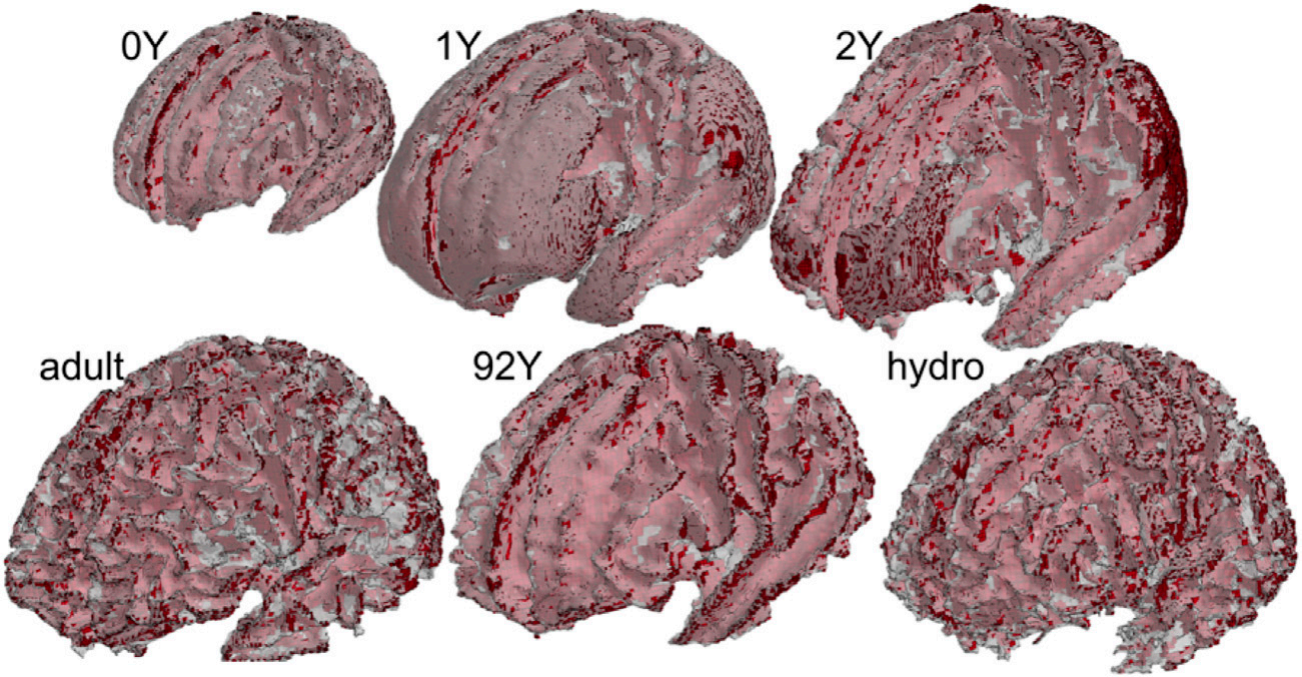
Regrouped WM based on subjects’ WM image mask for all the six subjects. The red color shows the WM elements, and the white transparent shows the surfaces reconstructed from the subject’s segmented WM image mask.

Figure 4 shows the regrouped WM elements of the morphed brain enclosed by the reconstructed surface of the segmented WM.

### Evaluation of Personalization Accuracy

To evaluate registration accuracy, the baseline ICBM image (i $mg_{baseline}$ ), which corresponds to the baseline head model, is warped via the inverse of displacement fields from each registration step ( $g_{subj}^{-1}$ ) (Eq. 5), resulting in a warped image ( $img_{warped}$ ) (Eq. 6).

$$g_{subj}^{-1}=g_{fn}^{-1}\left(g_{m2}^{-1}\left(g_{m1}^{-1}\left(g_{dram}^{-1}\left(g_{f2}^{-1}(g_{f1}^{-1}(g_{demo}^{-1})\right)\right)\right)\right),$$ (5)

$$img_{warped}=\;g_{fn}^{-1}\left(g_{m2}^{-1}(g_{m1}^{-1}(g_{dram}^{-1}(g_{f2}^{-1}(g_{f1}^{-1}\left(g_{demo}^{-1}\left(img_{baseline}\right)\right)))))\right).$$ (6)

DICE and 95th percentile Hausdorff distance (HD95) between the $img_{warped}$ and subjects’ images are then calculated to evaluate registration performance. As $img_{warped}$ corresponds to the personalized subject-specific model, both metrics also reflect the personalization accuracy of the generated subject-specific models.

To calculate DICE and HD95, automated segmentation is performed using the software FreeSurfer (version 7.1.0) with the default brain segmentation pipeline (*recon-all*) for both the warped ICBM and subjects’ T1W images. The segmented binary masks for the whole brain and local regions of cerebral GM, WM, CC, BS, hippocampus, thalamus, and cerebellum are used for DICE and HD95 calculation. For the cranial mask, the metrics are calculated based on manually segmented cranial by thresholding followed by noise removal. Similarly, one sagittal slice of CC is manually segmented and used to calculate both matrices. The use of manual segmentation for both regions is due to the insufficient quality (not reflecting the actual anatomy) by *recon-all* for the current dataset. Note that these segmented binary masks are only used for DICE and HD95 calculation, and the quality of the automatic segmentation has no influence on the subject-specific mesh development process.

#### DICE

DICE is a single metric to measure the spatial overlap between images defined as twice the number of elements common to both sets divided by the sum of the number of elements in each set (Ou et al., 2014):

$$DICE\left(A,B\right)=\frac{2\left|A\cap B\right|}{\left|A\right|+\left|B\right|},$$ (7)

where A and B denote the binary segmentation labels, |A| and |B| are the number of voxels in each set, and |A∩B| is the number of shared voxels by A and B . The DICE value of 0 implies no overlap between both, whereas a DICE coefficient of one indicates perfect overlap between the warped and the target image.

#### HD95

Hausdorff distance is defined as

HD(C,D)=max(h(C,D),h(D,C)), (8)

where C,D are the two sets of vertices from two segmented images

$$h\left(C,D\right)=\begin{array}{c} max\;\\ c\in C \end{array}\;\;\begin{array}{c} max\\ d\in D \end{array}c-d.$$ (9)

The 95^th^ percentile Hausdorff distance (HD95) is used following earlier studies (Ou et al., 2011; Ou et al., 2014). HD95 ranges from 0 to above; a lower value indicates a better registration accuracy between the warped and the target image.

### Application of the Framework for Subject-specific Model Generation

A complete registration pipeline is only needed for the most challenging case; fewer registration steps are sufficient for brains with small anatomical differences compared with the baseline. The following three typical subtypes of the pipeline are used to generate subject-specific models for the six subjects.

Type I This is the basic pipeline containing two steps: Demons registration of the cranial mask and Dramms registration of T1W image. This two-step pipeline has been shown to achieve good registration accuracy for six healthy adult subjects (Li et al., 2021). The capacity of this pipeline is further demonstrated with a 2Y brain.
Type II Multiple feature steps are added to the Type I pipeline, allowing align brains with significant anatomical changes. The capacity of this pipeline is demonstrated with a hydrocephalus brain using three feature steps.
Type III Multi-modality imaging registration steps are added to the Type I pipeline to improve brain alignment as demonstrated with an adult brain.

#### 2YO Model Generation via Pipeline Type I: Two Steps

**FIGURE 5:**
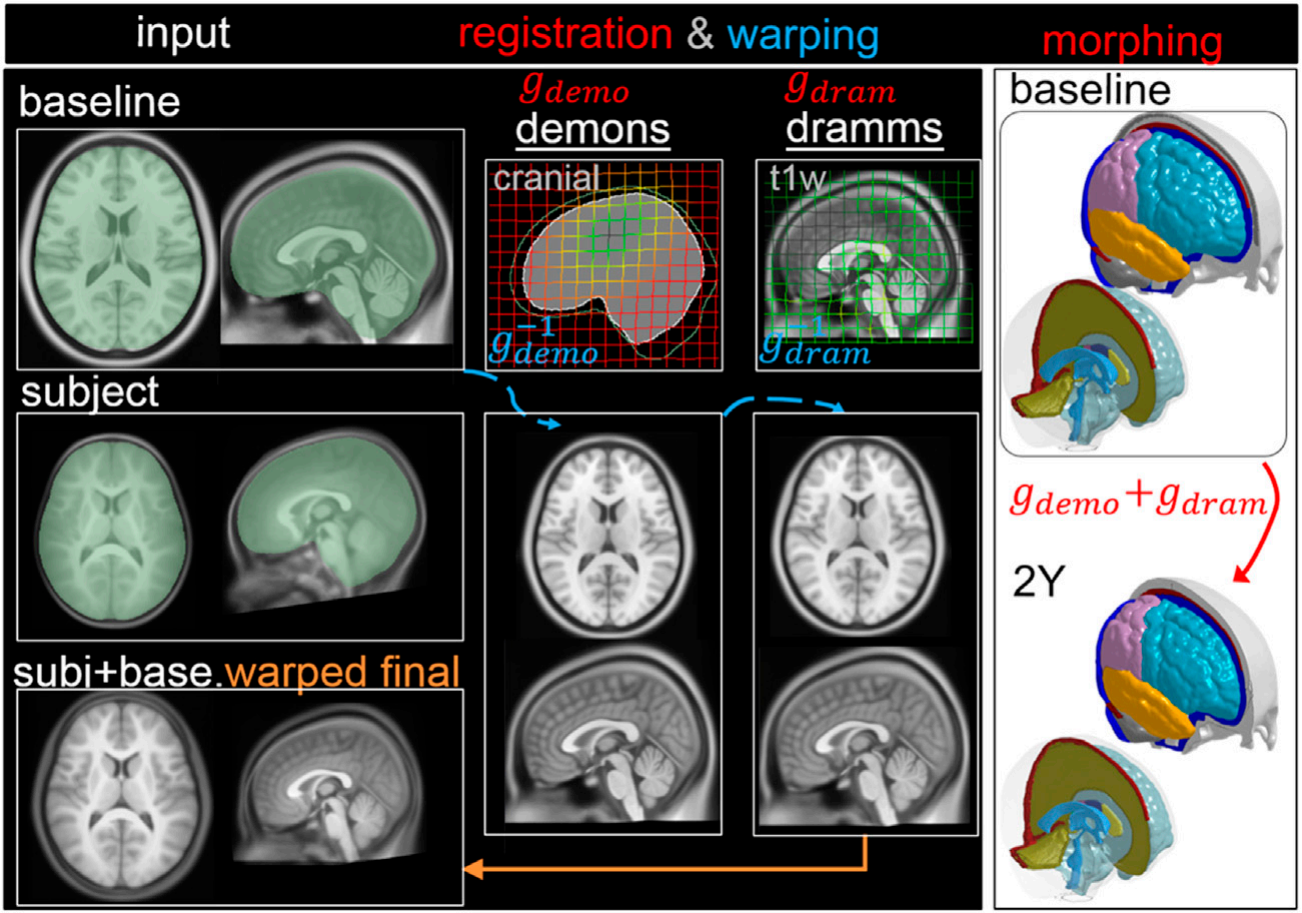
Type I pipeline applied for personalizing the baseline ADAPT model to a subject-specific model of a 2Y. The pipeline consists of two steps: (i) Demons registration with cranial masks; (ii) Dramms registration with T1W image. The displacement field obtained from each step is visualized on the grid together with the warped baseline ICBM images to show its effect. The final warped ICBM is overlaid with the subject’s image to visualize registration accuracy.

First, T1W images of ICBM and the 2Y brain are segmented to obtain the cranial masks, which are used as input for Demons registration, from which a dense displacement field $g_{demo}$ is obtained (Figure 5). Next, the baseline ICBM T1W image is warped by $g_{demo}^{-1}$ . The warped ICBM and subject’s T1W images are then skull stripped and serve as input for Dramms registration, obtaining $g_{dram}$ . The two displacement fields add up $g_{subj\_2YO}=g_{demo}+g_{dram}$ which is used to morph the baseline mesh, obtaining the subject-specific head model. Finally, the baseline ICBM image warped to the subject is obtained via $img_{warped\_2Y}=\;\left(g_{dram}^{-1}\left(g_{demo}^{-1}\left(img_{baseline}\right)\right)\right)$ , which is compared with the T1W image of the 2Y brain to evaluate personalization accuracy.

#### Hydrocephalus Model via Pipeline Type II: Multiple Features

**FIGURE 6:**
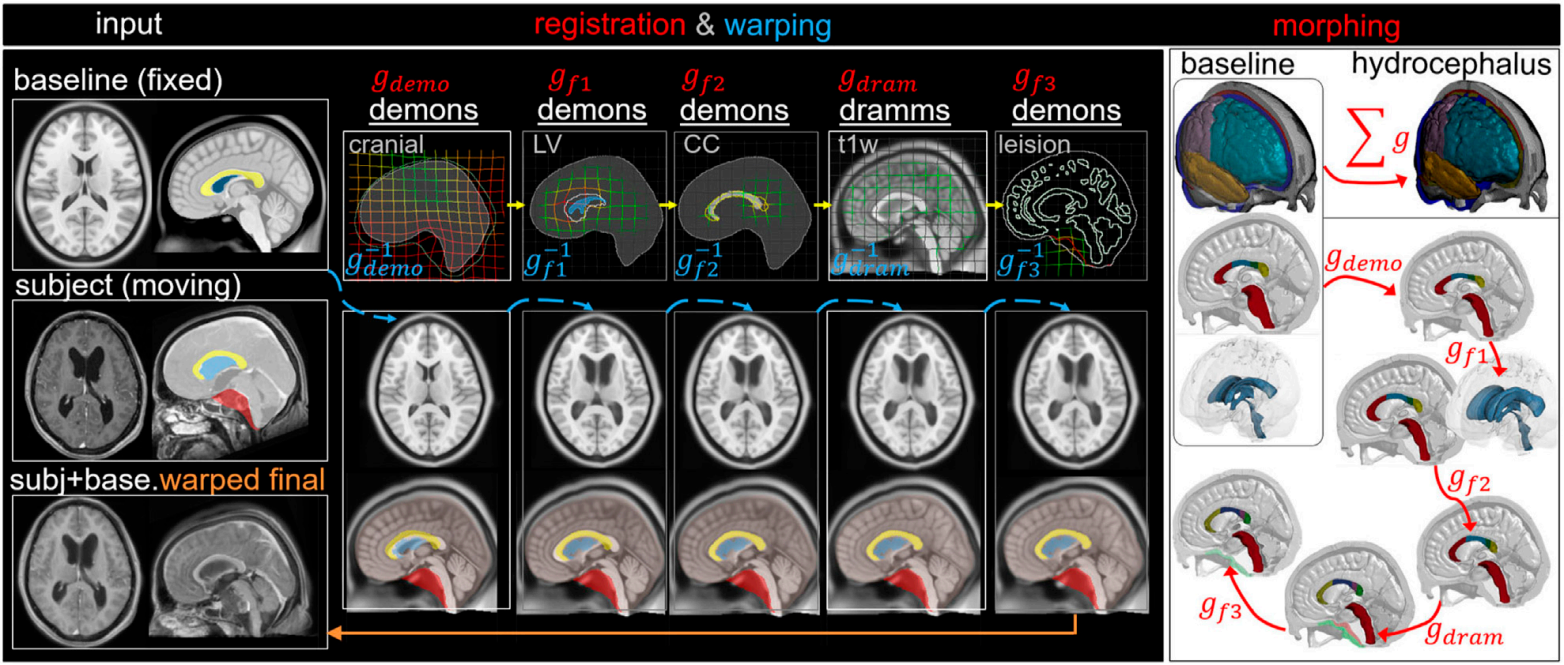
Type II pipeline applied for personalizing the baseline ADAPT model to a subject-specific model of a hydrocephalus brain. The pipeline consists of five steps: (i) Demons registration with cranial masks; (ii) Demons registration with segmented lateral ventricle (LV) mask for capturing the enlarged LV; (iii) Demons registration with segmented CC mask for capturing the CC shape; (iv) Dramms registration with T1W image for capturing local brain anatomy; (v) Demons registration to drag back the skull mesh which is pushed due to the lesion in the cranial mask in step (i). The displacement field obtained from each step is visualized on the grid together with the warped baseline ICBM images and morphed meshes to show its effect. The final warped ICBM is overlaid with the subject’s image to visualize registration accuracy.

The workflow is similar to the above, but three additional feature steps are added to capture the enlarged LV, deformed CC, and brain lesion, resulting in five dense displacement fields that add up as $g_{subj\_hydro}=g_{demo}+g_{f1}+g_{f2}+g_{dram}+g_{f3}$ , which is used to morph the baseline mesh and obtain a subject-specific head model (Figure 6). The baseline ICBM image warped to the subject is obtained via $img_{warped\_hydro}=\left(g_{dram}^{-1}(g_{f2}^{-1}(g_{f1}^{-1}\left(g_{demo}^{-1}\left(img_{baseline}\right)\right)))\right)$ , which is compared with the T1W image of the hydrocephalus subject to evaluate personalization accuracy.

#### Adult Brain Model via Pipeline Type III: Multimodality

**FIGURE 7:**
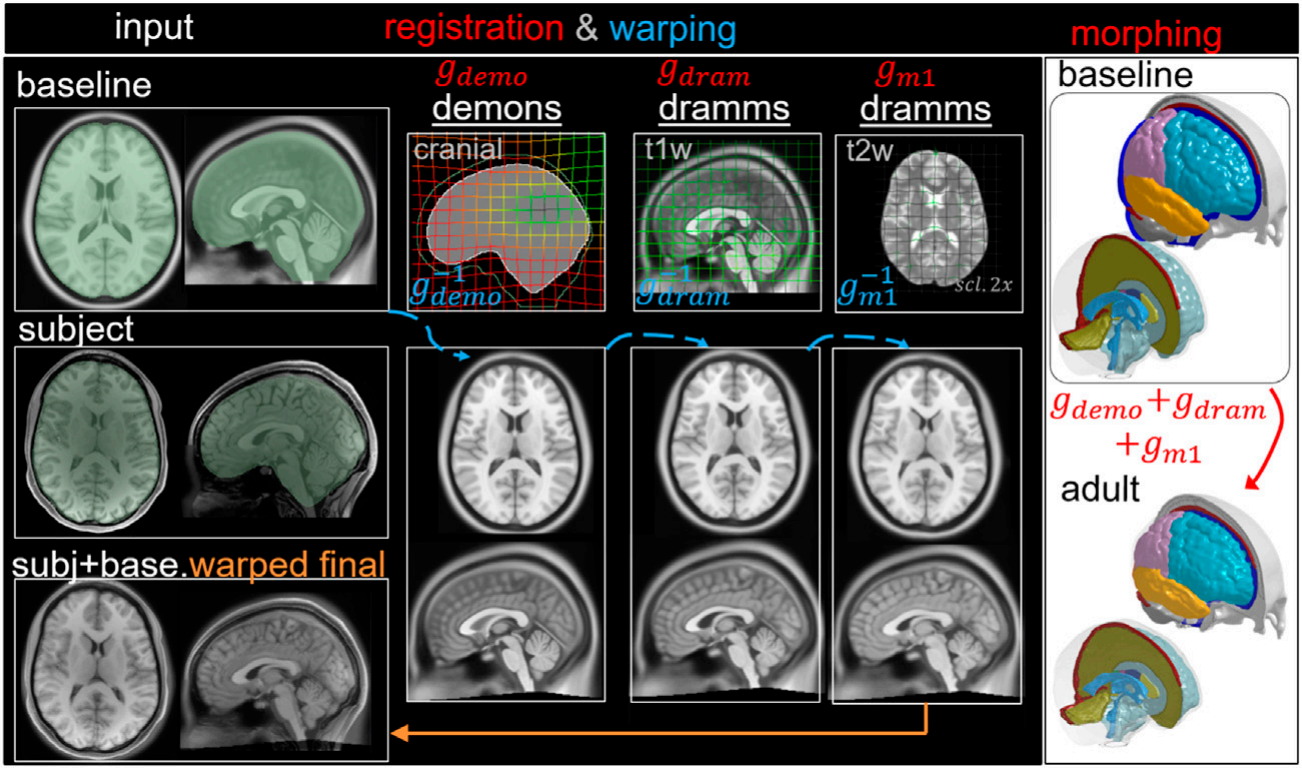
Type III pipeline applied for personalizing the baseline ADAPT model to a subject-specific model of an adult subject. The pipeline consists of three steps: (i) Demons registration with cranial masks; (ii) Dramms registration with T1W image; (iii) Dramms registration with T2W image for further alignment. The displacement field obtained from each step is visualized on the grid together with the warped baseline ICBM images to show its effect. The final warped ICBM is overlaid with the subject’s image to visualize registration accuracy.

The workflow is similar to Type I, but an additional multimodality T2W registration step is performed to further align the LVs resulting in three dense displacement fields added up as $g_{subj\_adult}=g_{demo}+g_{dram}+g_{m1}$ , which is used to morph the baseline mesh and obtain the subject-specific head model (Figure 7). The baseline ICBM image warped to the subject is obtained via $img_{warped\_adult}=\;g_{m1}^{-1}\left(g_{dram}^{-1}\left(g_{demo}^{-1}\left(img_{baseline}\right)\right)\right)$ , which is compared with the T1W image of the hydrocephalus subject to evaluate personalization accuracy.

#### Pipeline for the 0Y, 1Y, and the 92Y

The 92Y uses Type II pipeline, similar to the hydrocephalus subject, except only one feature step for LV is used, i.e., $g_{subj\_92Y}=g_{demo}+g_{f1}+g_{dram}$ . Interestingly, the Dramms registration captures the thinning of CC without the CC feature step as for the hydrocephalus subject. It could be due to the higher quality image of the 92Y than the hydrocephalus brain.

The 1Y could use the same pipeline as the 2Y. However, in this study, an alternative approach is used, using the 2Y as an intermediate step, i.e., align 1Y T1W (as *moving* image) to that of the 2Y (as *fixed* image) by Dramms registration, obtaining a displacement field $g_{1Y\_to\_2Y}$ . The final displacement field used for personalizing the baseline model to the 1Y writes: $g_{subj\_1Y}=$ $g_{subj\_2YO}+g_{1Y\_to\_2Y}$ , and the warped image is obtained via $img_{warped\_1Y}=\;\left(g_{1Y\_to\_2Y}^{-1}\left(g_{subj\_2Y}^{-1}\left(img_{baseline}\right)\right)\right)$ .

The 0Y uses the same pipeline as the 1Y by having 2Y as an intermediate step, i.e., align the 0Y T1W (as *moving* image) to that of the 2Y (as *fixed* image) by Dramms registration. Since the cerebellum for the 0Y was stripped in the original database (Figure 1A), a paired T1W image of the 2Y with cerebellum stripped (readily available in the database) is used for registration, from which a displacement field $g_{0Y\_to\_2Y}$ is obtained. The remaining steps for personalization and image warping are the same as for the 1Y described above. Note that although the registered images do not have cerebellum, the obtained displacement field $g_{0Y\_to\_2Y}$ defined in the entire image space does cover the cerebellum region despite values close to zero. The displacement field when used to morph the baseline model that has cerebellum, resulting in a final subject-specific model of the 0Y with cerebellum included.

## Results

### Subject-specific Head Models and Element Quality

**FIGURE 8:**
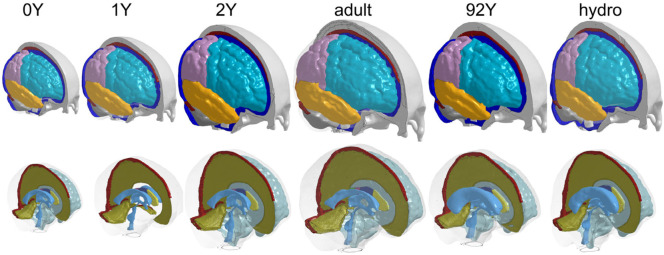
Six subject-specific head models generated including the 0Y, 1Y, 2Y, adult, 92Y, and a hydrocephalus brain (the same length scale applies).

**FIGURE 9:**
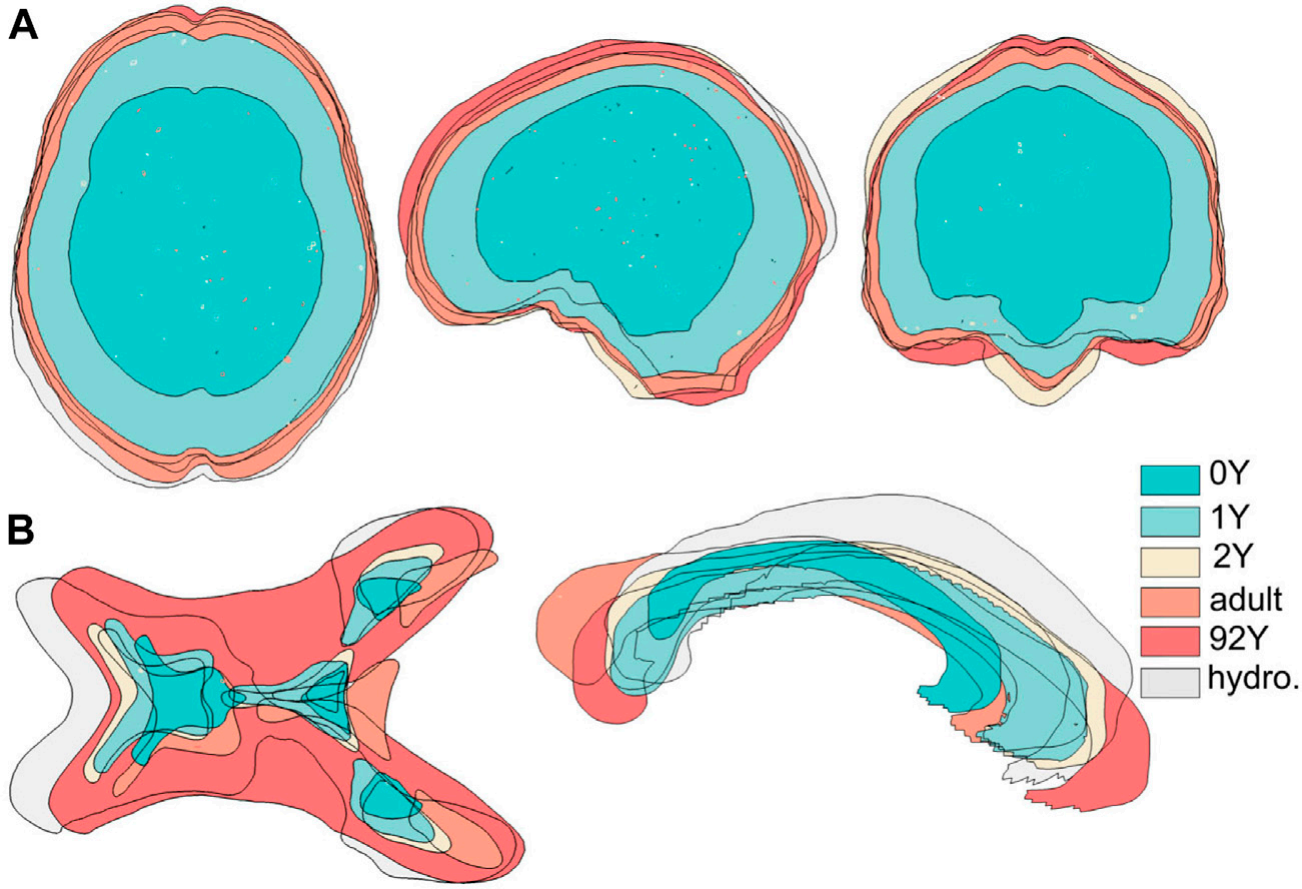
Six subject-specific models are aligned together, showing the generated models have widely varying intracranial volumes **(upper row)** and significant anatomical differences as exemplified with lateral ventricles and corpus callosum **(lower row)**.

**FIGURE 10:**
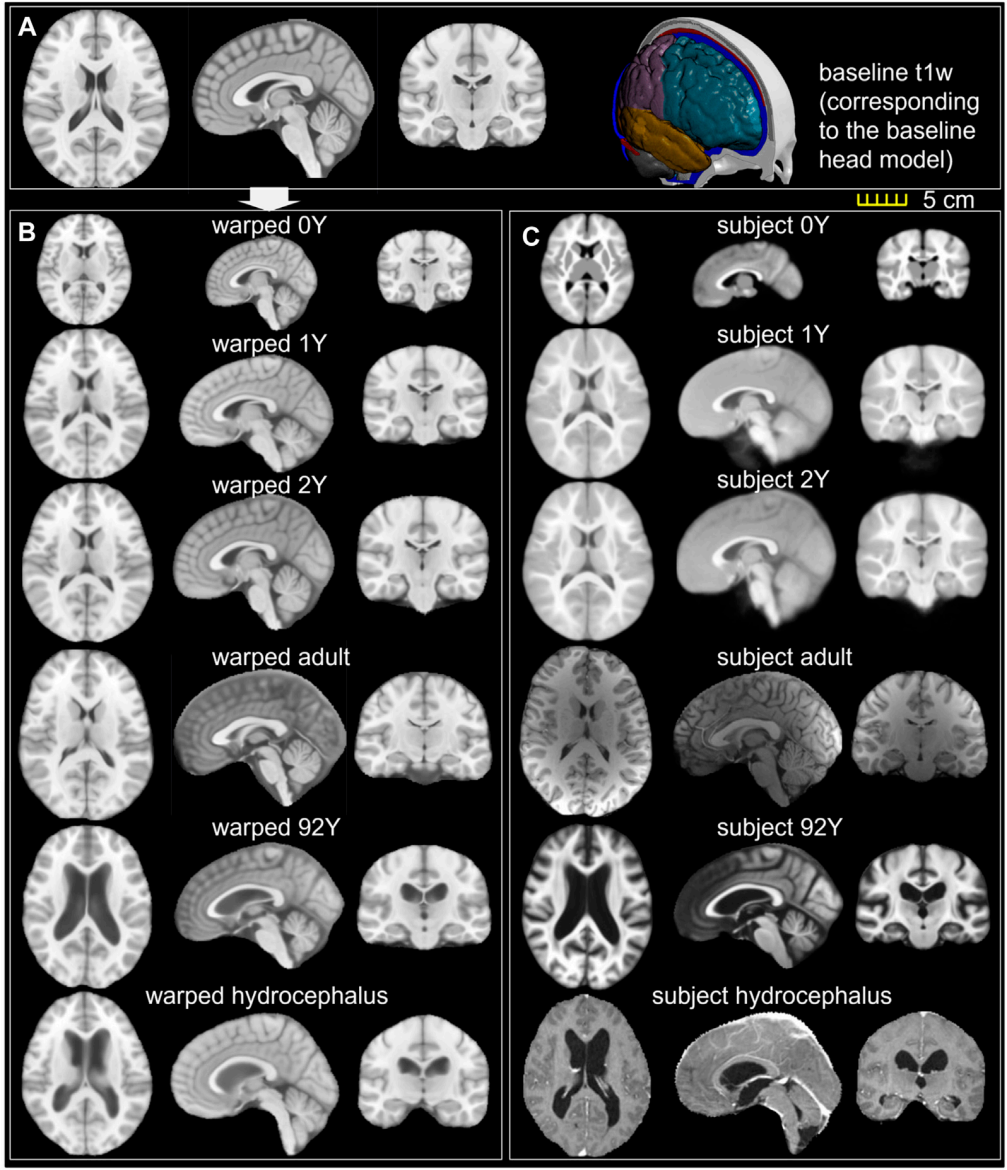
**(A)** T1W image of the ICBM baseline; **(B)** ICBM baseline warped to the six subjects; **(C)** T1W image of the six subjects. Transverse, sagittal, and coronal cross-sections are captured for each brain.

**FIGURE 11:**
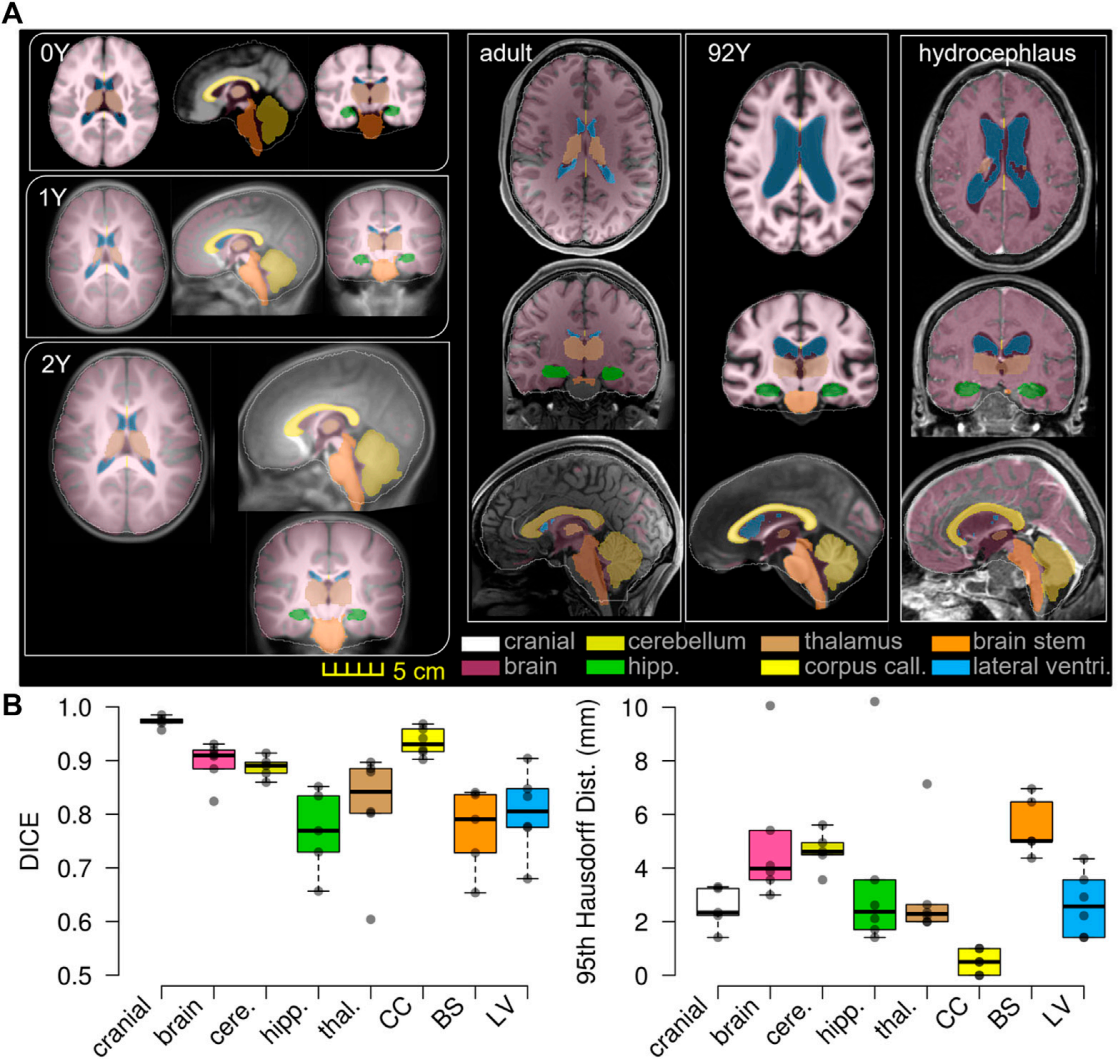
T1W image of the subject is overlaid with the segmented binary masks of the warped baseline, including the cranial mask, the brain, and local brain regions of the cerebellum, hippocampus, thalamus, CC, BS, and lateral ventricles **(A)**. Boxplots of DICE and 95HD. The boxplots show the median, minimum, and maximum values shown **(B)**.

The generated head models (Figure 8) and cross-sections (Figure 9) demonstrate the capacity of the framework for generating subject-specific head models with significant anatomical differences; all morphed from a baseline model. Especially, the extensively enlarged LVs and the varying shapes of CC in the hydrocephalus and the elderly 92Y brain are captured (Figures 10, 11). The element quality for the models is listed in Table 2, showing that most brain elements (95.9 ± 1.5% on average for the six subjects) have a Jacobian over 0.5, and the minimum Jacobian in all the six head models is above 0.13 (in the hydrocephalus brain). In this study, the mesh quality is considered satisfactory when at least 95% of the elements have a Jacobian over 0.5.

**TABLE 2:** Element quality of the baseline ADAPT model and the six subject-specific head models generated by morphing.

Head model	Element quality index											
	Jacobian ≥0.5		Warpage (°) ≤30		Skew (°) ≤60		Aspect ratio ≤8		Min. angle (°) ≥30		Max angle (°) ≤150	
	percent	min	percent	max	percent	max	percent	max	percent	min	percent	max
ADAPT	98%	0.45	92%	111.76	99.9%	69.95	99.9%	6.62	99.8%	17.98	99.9%	161.94
Personalized models												
0Y	96%	0.34	92%	112.60	99.9%	70.93	99.9%	7.99	99.9%	14.58	98.0%	168.41
1Y	97%	0.31	92%	111.99	99.9%	69.68	99.9%	8.20	99.9%	16.23	99.0%	167.85
2Y	97%	0.36	92%	110.26	99.9%	67.19	99.9%	6.64	99.9%	16.39	99.0%	166.38
Adult	93%	0.17	90%	139.66	99.9%	74.43	99.9%	10.09	99.0%	8.95	98%	215.91
92Y	96%	0.15	94%	121.81	99.9%	78.53	99.9%	16.09	99.0%	5.56	98%	177.29
Hydrocephalus	95%	0.13	91%	118.31	99.9%	77.71	99.9%	11.07	99.0%	7.80	97%	176.79

### Personalization Accuracy

The baseline ICBM image (Figure 10A) is warped to the six subjects. The warped images (Figure 10B) and subjects’ images (Figure 10C) are compared to evaluate registration accuracy. The segmented binary masks of the final warped baseline and subjects are overlaid to further visualize personalization accuracy (Figure 11A). The evaluated masks include cranial, brain, and six local brain regions. The boxplots of the DICE and HD95 are presented in Figure 11, with values listed in Tables 3, 4. The average DICE scores are all >0.9 for the cranial mask, the brain, cerebellum, CC, being 0.97, 0.90, 0.89, and 0.94, respectively. Since the cerebellum for the 0Y subject image has been stripped, the evaluation of registration accuracy is without this region. The average DICE score for LV is 0.80. DICE score can be improved by incorporating multimodality step, e.g., with T2W image that has higher contrast for CSF/LVs. For example, the pipeline for the adult subject adding the T2W multimodality step improves personalization accuracy than previously achieved (see Supplementary Appendix S1). The DICE values are comparable to that achieved in neuroimaging field (Ou et al., 2014) despite the higher requirement on the smoothness of displacement field for satisfactory element quality in the personalized head FE models.

**TABLE 3:** DICE coefficients for the six subjects.

Subject ID	Cranial	Brain	Cerebellum	Hippocampus	Thalamus	CC	BS	LV
0Y	NaN	0.82	NaN	NaN	0.60	0.92	NaN	0.78
1Y	0.96	0.93	0.89	0.83	0.89	0.96	0.79	0.85
2Y	0.98	0.92	0.90	0.85	0.90	0.94	0.84	0.83
Adult	0.97	0.91	0.88	0.77	0.88	0.90	0.73	0.68
92Y	0.97	0.91	0.91	0.73	0.80	0.97	0.84	0.90
Hydrocephalus	0.99	0.88	0.86	0.66	0.80	0.92	0.65	0.78
Average	0.97	0.90	0.89	0.77	0.81	0.94	0.77	0.80

**TABLE 4:** HD95 for the six subjects.

Subject ID	Cranial	Brain	Cerebellum	Hippocampus	Thalamus	CC	BS	LV
0Y	NaN	10.06	NaN	10.21	7.14	1.0	NaN	2.22
1Y	3.24	5.41	4.5	1.71	2.24	0	5.01	1.41
2Y	2.34	4.10	4.61	1.41	2.0	0.5	4.37	1.41
Adult	3.3	3.56	4.95	2.12	2.0	1	5.00	2.92
92Y	2.24	3.0	3.56	2.62	2.34	0	6.96	3.56
Hydrocephalus	1.41	3.87	5.61	3.56	2.64	0.5	6.47	4.35
Average	2.51	5.0	4.65	3.61	3.06	0.50	5.56	2.65

### Hydrocephalus and the Elderly Brain: Importance of the Feature Step and the Higher Requirement on Displacement Smoothness for Mesh Morphing

**FIGURE 12:**
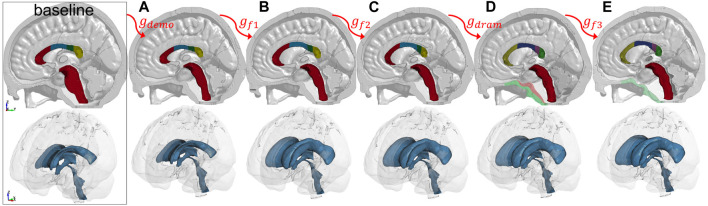
Morphed FE meshes after each of the five steps for the hydrocephalus subject.

The mesh after each morphing step shown in Figure 12 illustrates the effect of the feature steps, which allow capturing subject’s cranial shape (Figure 12A), enlarged LVs (Figure 12B), CC (Figure 12C), as well as pushing back of the skull mesh (Figure 12E), while local brain structures are captured by Dramms registration (Figure 12D). The meshes from these intermediate steps are morphed from the baseline ADAPT head model with displacement fields obtained via the image registration pipeline shown in Figure 6.

**FIGURE 13:**
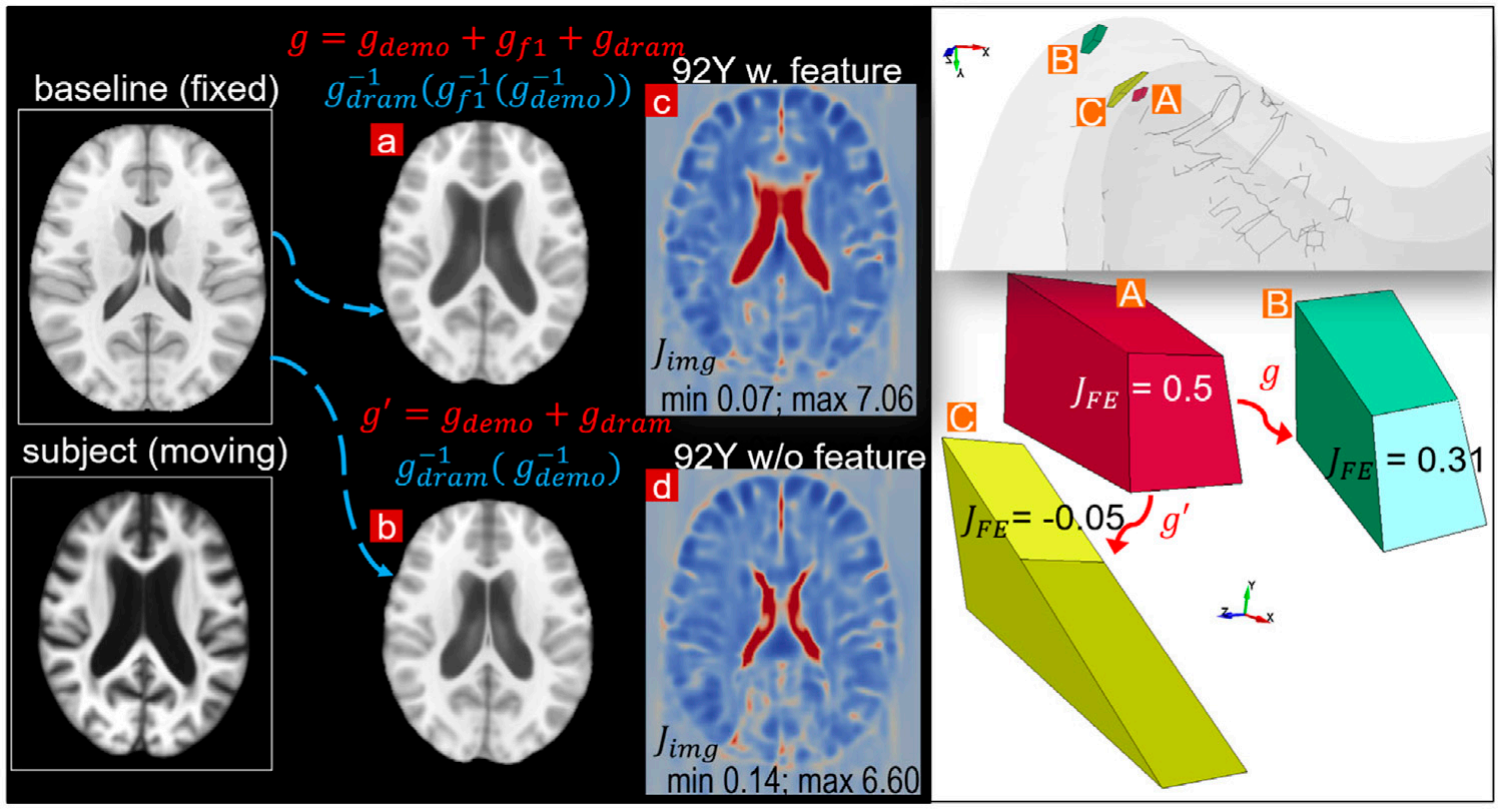
Parametric pipeline for the 92Y without the LV feature step compared with the default pipeline. The warped baseline image by the parametric pipeline **(B)** does not capture the enlarged LV compared with that achieved by the default pipeline **(A)**. The parametric pipeline leads to negative Jacobian in some elements in the personalized mesh **(right figure)**, although the Jacobian map ( $J_{img}$ ) of the final obtained displacement field is all positive. One representative axial slice is shown with the minimum and the maximum value of $J_{img}$ in the entire brain indicated **(C,D)**.

To further illustrate the importance of the feature registrations, a parametric pipeline without the LV feature step is performed for the 92Y brain, i.e., a complete pipeline writes $g_{subj\_92Y}=g_{demo}+g_{dram}$ and $img_{warped\_92Y}=\;g_{dram}^{-1}(g_{demo}^{-1}\left(img_{baseline}\right))$ (Figure 13). The results show that the Dramms registration, even using the largest allowable smoothness weight (*g* = 1), leads to FE mesh with negative Jacobian in some elements. For example, one FE element at the frontal horn of the LVs with a Jacobian ( $J_{FE}=$ 0.5) in the baseline mesh (Figure 13A, right upper), when morphed by the parametric pipeline resulting in a negative Jacobian ( $J_{FE}=$ -0.05) (Figure 13C, right upper). In contrast, when morphed by the original three-step pipeline, the same element has a positive value ( $J_{FE}=$ 0.31) (Figure 13B, right upper). This parametric pipeline also results in lower registration accuracy than the three-step pipeline (Figures 13A,B). This example also demonstrates the higher requirement on the smoothness of displacement than in the neuroimaging field when only Jacobian of the displacement field ( $J_{img}$ ) is of concern (more detailed analysis presented in Supplementary Appendix S2).

## Discussions

This study presents a personalization framework for the efficient generation of subject-specific head models. The framework consists of hierarchical multiple feature and multimodality imaging registration pipelines, mesh morphing, and mesh grouping. The registration pipeline achieves competitive registration accuracy despite a higher requirement on the smoothness of the displacement field concerning the element quality of the morphed mesh. The Demons feature registration steps capture significant anatomical differences, allowing a good initialization before applying Dramms registration to further capture the inter-subject anatomical details. The Dramms registration step with multimodality imaging further improves brain alignment. As a final step of the framework, mesh grouping of WM according to the subject’s image mask allows incorporating subject-specific WM directly. The framework is successfully applied to subjects across the lifespan and a hydrocephalus brain with significant anatomical changes, achieving competitive personalization accuracy. The results demonstrate that the framework can personalize the baseline head model to brains with significant anatomical differences, resulting in subject-specific models ready for personalized simulations without manual repairing. To the knowledge of the author, this is the first study aligning such a broad scope of brain images suitable for mesh morphing.

The efficiency of the hierarchical two-step pipeline combining Demons and Dramms (Type I) has been previously assessed with six healthy adult subjects that have high-quality T1W images (Li et al., 2021). In this study, an extended pipeline is proposed for obtaining high-quality alignment across heterogeneous data of lifespan and for pathological brains with significant anatomical changes by introducing multiple feature steps as demonstrated with the hydrocephalus (Figure 12) and the 92Y brain (Figure 13). The registration accuracy for these more challenging cases is comparable with the six healthy adults, with average DICE scores for the cerebellum, CC, and brain all above 0.89. Notably, the average DICE score for LV for the six subjects in this study is 0.80, higher than that of the six adult subjects (0.71) (Li et al., 2021). Note that the same adult subject in an early study (Li et al., 2021) (subject ID 771354) is used here by adding T2W multimodality registration step (Type III). T2W images with higher contrast for CSF/LVs improve personalization accuracy compared with previous results (see Supplementary Appendix S1). The mesh grouping step incorporates subject-specific WM directly, which is important for infant models to accurately capture the rapid transition between GM and WM in early infancy. Thus, the promising performance demonstrates the potential of the framework to personalize the baseline model to almost any brains with significant anatomical changes. Besides hydrocephalus, personalized models for brains with other structural changes such as decompressive craniotomy with brain expanded outside the skull (Holst et al., 2012) can also be achieved.

Inter-subject registration between brains with significant anatomical differences is still challenging in neuroimaging field and has limited registration accuracy (Kim et al., 2015); even more challenging is to apply image registration for mesh morphing due to the higher requirement on the smoothness of the obtained displacement fields concerning element quality of the morphed mesh. There is often a trade-off between registration accuracy and element quality, and higher registration accuracy tends to worsen element quality according to the experience with the six subjects in this study. Especially, FE elements become invalid if their Jacobian become negative, which are not accepted by most FE analysis software. While in neuroimaging field, for physically plausible morphing, only positive Jacobian determinant of displacement field is to be ensured, which is often a looser requirement than FE Jacobian (see detailed analysis in Supplementary Appendix S1). Despite the higher requirement, this study achieves competitive registration accuracy compared with that reported in the neuroimaging field (Ou et al., 2014). For example, a previous study reported Jaccard index below 0.6 for all brain regions using popular deformable registration algorithms for inter-subject registration (Ou et al., 2014), while the average Jaccard index (converted from DICE according to Jaccard index = DICE/(2-DICE) (Ou et al., 2014)) for the six subjects is all above 0.66 for all regions in this study.

The applications of the framework show that different pipelines can be used depending on the anatomical differences between the subject and baseline ICBM, as well as the subject’s image quality and available imaging modalities. For brains that are similar to the baseline, Type I pipeline with fewer steps is sufficient, while for brains with significant anatomical differences compared with the baseline, e.g., the hydrocephalus and elderly brain, Type III pipeline is needed to achieve a proper alignment. Furthermore, when T2W images are available, multimodality image allows better alignment of the brain and CSF/LVs. In principle, more multimodality registrations can be performed if available from the subject as the baseline image contains imaging modalities of T1W, T2W, proton density (PD), and tissue probability maps. Besides, more feature steps can be introduced to handle even more challenging cases. Choosing the proper pipeline for a specific case needs trial and error. An overall guideline is to start from Type I then add more registration steps if needed. Note that the multiple feature steps can be combined into one image with multiple binary masks and perform Demons registration at once. However, one feature in each step, as done in this study, tends to be more robust. Furthermore, the framework, though demonstrated with the ADAPT baseline head model, is equally applicable for personalizing other head models as a baseline, e.g., models with tetrahedral elements as commonly used for tDCS, TMS, TUS, as well as smoothed-voxel brain models.

Compared with existing studies registering adult brains, fewer studies align infant brains, which are more challenging partially due to the rapid development of brain anatomy within the first year, especially T1W images are inversed with densities. Not only more challenging for registration algorithms but also the evaluation of performance is also more difficult as most segmentation algorithms are developed based on adult images, such as FreeSurfer. It’s worth noting that the lowest registration accuracy in all brains is for the thalamus in the 0Y; a visual check shows FreeSurfer automatically segmented thalamus not accurate enough. Future studies can employ infant Freesurfer (Zöllei et al., 2020) for more accurate segmentation for infant brain images, thus allows more objective evaluation of personalization accuracy. For adult brain mesh morphing, image registration-based morphing pipelines proposed earlier show promising performance in generating detailed subject-specific head models of healthy adult brains (Giudice et al., 2020; Li et al., 2021) while the framework proposed in this study allows generating models across the lifespan and for brains with significant anatomical changes, which can be used for studying age-specific and groupwise TBIs. Especially, brains with neurological diseases such as hydrocephalus with extensively enlarged LVs mimicking the elderly brain may provide a possible clue for new insights into TBIs. The approach also opens the opportunity for studying how a potentially vulnerable brain, e.g., a hydrocephalus patient, may sustain a TBI injury risk under fall impact, especially hydrocephalus patients who are more prone to fall. Until today, the biomechanics of TBIs in these groups are much understudied, partially due to the meshing challenge.

Compared with the many existing studies of TBIs for healthy adults, the injury mechanisms of infants and children are understudied. There are few child/infant head models (Li et al., 2011; Giordano et al., 2017; Li et al., 2017). In addition to the meshing challenge for adult models, the development of additional unique features of suture and fontanel plays an essential role in head impact response (Li et al., 2017). Previously, mesh morphing has also been used for morphing a baseline infant head model to different ages using radial basis function (RBF) to interpolate the displacement field obtained from land markers the anatomical features of suture and skull surface (Li et al., 2011). Unlike the image registration-based morphing, the RBF approach needs manual indentation of land markers, which is often tedious (Wu et al., 2019). The RBF approach also does not account for brain anatomies. Comparatively, the morphed detailed infant brain models in this study, when combined with the detailed skull and scalp models (Li et al., 2017; Li et al., 2019), will allow studying brain injury biomechanics under impact for infant head model for abusive head trauma with important legal applications for forensic diagnosis. The newborn infant head models may be used for studying delivery-related neurotrauma and studying new intervention approaches for clinical problems.

Some limitations and future works need to be mentioned here. First, the proposed framework allows efficient generation of subject-specific head models with competitive personalization accuracy and satisfactory element quality without mesh repairing. However, the morphing technique involves manual intervention when selecting which morphing pipeline to use. Thus, there could be user-to-user variability based on which pipelines are chosen and concerns regarding repeatability. For example, selecting improper pipelines could result in reduced morphing accuracy, and certain regions may not be morphed accurately if they are not selected as features by the user. Secondly, this framework requires segmentation for the Demons steps, which may need manual effort to ensure accurate segmentation and would require significant time and effort for large-scale studies. Nevertheless, considering the challenge of generating subject-specific head models, this effort is considered acceptable. Note that this morphing technique generates subject-specific models from a geometric perspective only and does not account for subject-specific material properties. Thirdly, the current framework allows generating head models reflecting the subject’s internal brain structures, but the major sulci and gyri lines are not evaluated like most studies in the neuroimaging field. It should also be noted that the framework does not ensure the same characteristic lengths among generated models of different sizes; the infant brains, in general, have smaller elements than that of an adult model. In this regard, the block-based method has an advantage that allows adjusting mesh densities to maintain similar element characteristic lengths (Mao et al., 2013b). Furthermore, Dramms registration algorithm is chosen for registering brain MRI images in this study since it has a clear advantage to align largely different anatomies such as the ventricles in comparison with other popular registration algorithms (Ou et al., 2014). However, other algorithms, such as implemented in ANTs (Avants et al., 2011) and DARTEL (Vercauteren et al., 2009), when used within the current framework, may achieve similar performance, but it is yet to be investigated. Finally, the framework can be extended to include more registration steps and other advanced nonlinear registration algorithms to handle even more challenging cases.

## Data Availability

The original contributions presented in the study are included in the article/Supplementary Material; further inquiries can be directed to the corresponding author.
