# Supplementary material for: Subject-Specific Head Model Generation by Mesh Morphing: A Personalization Framework and Its Applications
Source: Front Bioeng Biotechnol. 2021 Oct 18;9:706566. doi: 10.3389/fbioe.2021.706566 (PMC8558307; doi:10.3389/fbioe.2021.706566)
Supplement: Supplementary file 1 [file Presentation1.pdf]

Supplementary Material

Subject-Specific Head Model Generation by Mesh Morphing: A Personalization Framework and Its Applications

Xiaogai Li *
Division of Neuronic Engineering, Department of Biomedical Engineering and Health Systems, KTH Royal Institute of Technology, Stockholm, Sweden

*Correspondence: Xiaogai Li (xiaogai@kth.se)

Supplementary Appendix S1: Adult subject with multimodality registration

Multimodality T2W Dramms registration improves registration accuracy for local brain regions as demonstrated with the adult subject, especially the DICE score for LV increases from 0.62 to 0.68 (**Figure A1**).

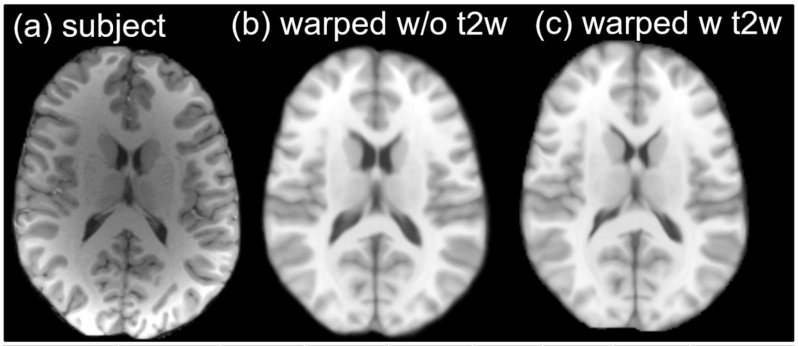

DICE	cranial	brain	cere.	hipp.	thal.	CC	BS	LV
w/o t2w	0.97	0.89	0.87	0.68	0.86	0.90	0.69	0.62
w t2w	0.97	0.91	0.88	0.77	0.88	0.90	0.73	0.68

Figure A1 An axial slice of the subject's image **(a)** is compared with the warped images obtained without T2W registration step **(b)** and that with the T2W step **(c)**, showing improved registration accuracy, especially for the LVs.

Supplementary Appendix S2: Higher requirement on displacement smoothness for mesh morphing than neuroimaging field

The terminology Jacobian is used in both FE modeling (FEM) and neuroimaging field. In FEM, Jacobian (J_{FE}) (**Eqn. A1**) describes element quality and indicates how an element defined in global coordinate system (**X**) derivates from ideal shape defined in the natural coordinate system (**r**), defined as [1]:

$$\frac{\partial}{\partial \mathbf{r}} = \mathbf{J}\frac{\partial}{\partial \mathbf{X}}$$
$$J_{FE} = \det(\mathbf{J}) \qquad \text{(A1)}$$

Where **X** and **r** denote the global and natural coordinate system, respectively. **J** is the Jacobian matrix, and J_{FE} is the determinant, which is scalar and is often abbreviated as Jacobian. J_{FE} measures the derivation of an element from its ideal shape, such as hexahedron's derivation from a cubic hexahedral element. J_{FE} ranges from -1.0 to 1.0, where 1.0 represents a perfectly shaped element, e.g., a cubic hexahedral element has $J_{FE} = 1$. Elements with $J_{FE} < 0$ have concave shapes, which are not accepted by most FE software. The values of J_{FE} for some representative elements are illustrated in **Figure 13**.

Jacobian in neuroimaging field is defined as:

$$J_{img} = \det\left(\mathbf{I} + \frac{\partial \mathbf{u}}{\partial \mathbf{X}}\right) \qquad \text{(A2)}$$

Where **X** is the global coordinate system, **I** is unit tensor, **u** is the displacement field obtained from image registration. J_{img} describes the volumetric change at a voxel. $J_{img} > 0$ indicates one-to-one mapping between the *fixed* and *moving* images, which is physically plausible. **Figure A2** shows that the displacement fields used for mesh morphing all have $J_{img} > 0$, indicating physically plausible displacement fields have been obtained from the registration pipelines.

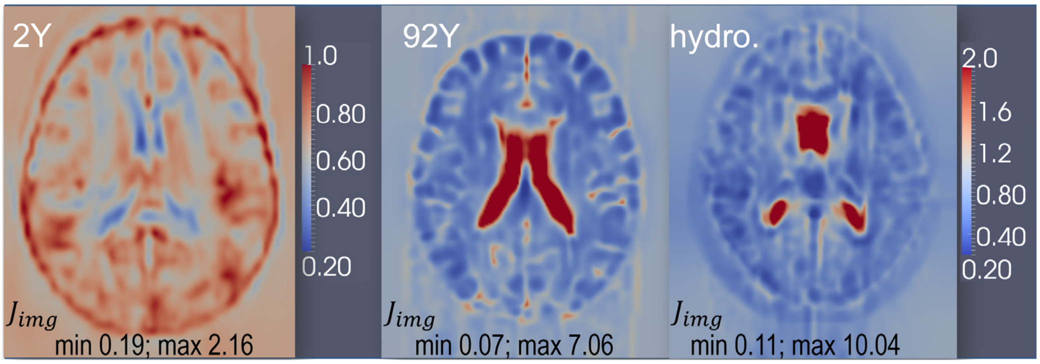

Figure A2 Positive Jacobian map of the final displacement field obtained from registration pipelines demonstrated with the 2Y, 92Y, and the hydrocephalus brain. One representative axial slice is shown with the minimum and maximum values of J_{img} in the entire brain indicated.

Hexahedral elements usually derivate from the ideal shape and have $J_{FE} < 1$. Thus, when applying **u** to morph already non-ideal shaped hexahedral elements, it could lead to elements with negative J_{FE} ,

although the Jacobian map (J_{img}) of $\mathbf{u}$ is all positive. For example, the displacement field obtained from the parametric pipeline though has positive J_{img} (**Figure 13D**) leads to element with negative J_{FE}. Therefore, there is a higher requirement on the smoothness of the obtained displacement field when used for mesh morphing (requiring both J_{FE} and J_{img} to be positive) than that in the neuroimaging field, which only require J_{img} to be positive for physically plausible one-to-one mapping between registered images.

References

[1] Bathe, K. J. (2006). Finite Element Procedures. Prentice-Hall, Englewood Cliffs, New Jersey.
